# Orelabrutinib and rituximab regimen combined with intravitreal methotrexate for treatment of primary vitreoretinal lymphoma: a case report and literature review

**DOI:** 10.3389/fonc.2026.1800529

**Published:** 2026-04-02

**Authors:** Yuxin Li, Ruiyun Qiao, Xuan Zhang, Yahong Li

**Affiliations:** Department of Hematology, Xi’an People’s Hospital (Xi’an Fourth Hospital), Xi’an, Shaanxi, China

**Keywords:** Bruton tyrosine kinase, orelabrutinib, primary central nervous system lymphoma, rituximab, targeted therapy

## Abstract

Primary vitreoretinal lymphoma (PVRL) is a rare intraocular malignancy for which the optimal therapeutic strategy remains controversial, largely due to the disease’s rarity and considerable heterogeneity in treatment approaches across medical centers. While some studies suggest that combined systemic chemotherapy may prevent central nervous system progression in PVRL, others have failed to confirm such benefits, particularly given the severe treatment-related toxicities associated with intensive regimens. Although agents such as lenalidomide and Bruton’s tyrosine kinase(BTK) inhibitors have demonstrated efficacy in relapsed/refractory (R/R)PVRL, their role in treatment-naïve patients remains unclear. Herein, we retrospectively report the efficacy and safety of Orelabrutinib and rituximab combined with intravitreal methotrexate(MTX) in a patient with PVRL. The patient received this regimen as first-line treatment, which led to rapid improvement in visual acuity and intraocular tumor control in all affected eyes. Interleukin-10, a well-established biomarker for vitreoretinal lymphoma, decreased to normal levels after five months of therapy. The treatment was well-tolerated, with no reported adverse events. In conclusion, the combination of Orelabrutinib, rituximab, and intravitreal MTX is a feasible therapeutic strategy for PVRL. Our findings may contribute to a potential paradigm shift in the management of this rare disease.

## Introduction

Primary vitreoretinal lymphoma (PVRL) is a rare subtype of primary central nervous system lymphoma (PCNSL),approximately 95% of which are diffuse large B-cell lymphomas (DLBCL) (1–4). In the United States, the overall age-adjusted incidence of PVRL was 0.23 per 1,000,000 person-years (5). Patients with PVRL have a poor overall prognosis. Patients with isolated ocular or CNS involvement have a relatively better outcome. In contrast, those who develop CNS involvement have a particularly dismal prognosis, with a median progression-free survival of only 3.5 months (6, 7).

To date, no standard treatment approaches have been defined. The current therapeutic options are comprised of ocular treatments, systemic treatments or a combination of both. While local ocular treatment, such as intravitreal methotrexate(MTX) and ocular radiotherapy, effectively control intraocular disease, they are associated with a high rate of CNS relapse and various treatment-related toxicities. Systemic treatment options primarily includes chemotherapy, generally centered on high-dose MTX, and novel targeted therapy, chiefly rituximab and Bruton’s tyrosine kinase (BTK) inhibitors such as ibrutinib and zanubrutinib. Ibrutinib is effective for r/r PCNSL but limited by off-target toxicities. Orelabrutinib, a novel highly selective second-generation BTK inhibitor, exhibits superior blood-brain barrier permeability, favorable bioavailability, and excellent safety profiles with minimal off-target effects (8–10).The therapeutic regimen combining zanubrutinib, rituximab, and intravitreal methotrexate in PVRL patients necessitates further investigation with larger sample sizes. This study aims to address this gap by evaluating the safety and efficacy of Orelabrutinib and Rituximab combination through clinical case analysis.

## Case report

**Figure 1:**
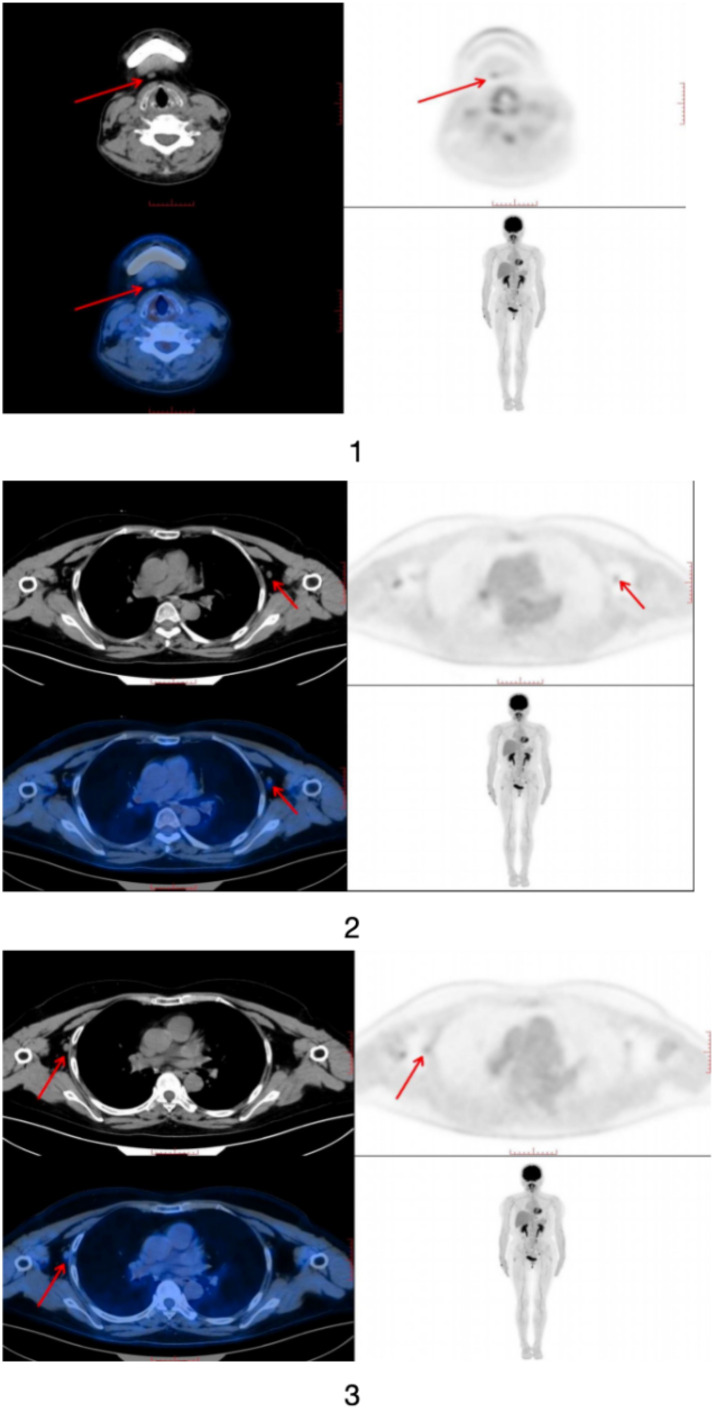
PET/CT images of the patient.

A 55-year-old female patient presented to the Ophthalmology Department of Xi’an People’s Hospital in February 2025, complaining of “blurred vision in both eyes, persistent in the left eye for over 5 months and newly onset in the right eye for 1 day.” Bilateral vitreous aspiration was performed sequentially. Vitreous fluid analysis revealed an IL-10/IL-6 ratio of 39.5644, with IL-6 at 90.9 pg/mL and IL-10 markedly elevated to 3596.4 pg/mL. Cytological examination of the vitreous samples showed scattered atypical small round cells with significant degeneration, suggestive of a neoplastic process. To further clarify the diagnosis, the patient underwent lumbar puncture on March 11, 2025. Cerebrospinal fluid analysis showed an IL-10/IL-6 ratio of 36.6667, with IL-10 at 11 pg/mL and IL-6 at 0.3 pg/mL. Whole-body PET/CT imaging revealed no abnormal hypermetabolic foci in the globes or brain parenchyma (**Figure 1**); however, small lymph nodes with mildly increased radionuclide uptake were observed in the cervical and axillary regions. Subsequently, the patient received bilateral intravitreal methotrexate injections and was transferred to the Hematology Department for further evaluation and management.

In the Hematology Department, bone marrow aspiration and flow cytometry of the cerebrospinal fluid showed no abnormalities. Brain MRI indicated possible posterior scleral staphyloma in both eyes and abnormal subcutaneous signals in the bilateral maxillofacial and frontotemporal regions. A clinical diagnosis of primary vitreoretinal lymphoma (PVRL) with central nervous system involvement was established. However, due to insufficient cellular material from the intraocular specimens, further testing such as genetic mutation analysis, flow cytometry, and immunohistochemistry could not be performed. Given the patient’s methotrexate gene polymorphism test results indicating mild abnormalities, she declined high-dose methotrexate therapy. A treatment regimen consisting of rituximab combined with oral orelabrutinib, supplemented with intravitreal methotrexate injections, was initiated. An induction therapy regimen of 6 cycles was administered: rituximab 375 mg/m² intravenously once per cycle plus oral orelabrutinib 150mg once daily, and intravitreal methotrexate (400 μg) injections.

**Figure 2:**
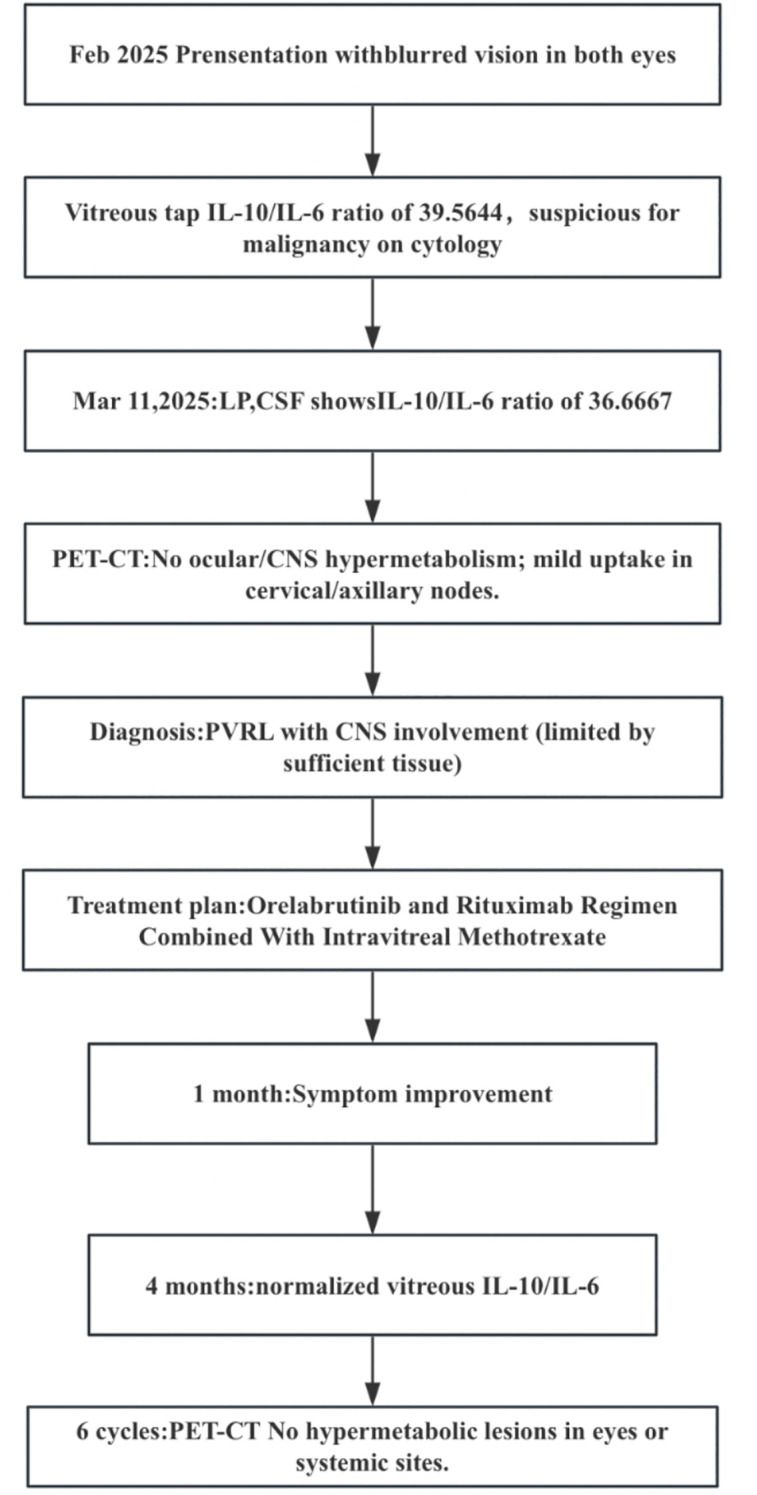
Diagnostic and therapeutic flowchart of a patient with PVRL.

One month after treatment initiation, the patient’s blurred vision significantly improved. Re-examination at 4 months showed that IL-10, IL-6, and the IL-10/IL-6 ratio in the vitreous fluid had returned to normal ranges. No adverse effects such as myelosuppression, hemorrhage, diarrhea, arthritis, or atrial fibrillation were observed during the entire treatment course. After completing six cycles of rituximab combined with orelabrutinib, follow-up PET/CT imaging showed no abnormal hypermetabolic foci in the globes or other systemic regions. The patient’s clinical course is summarized in **Figure 2**.

## Discussion

PVRL usually masquerades as uveitis, leading to frequent misdiagnosis as recurrent chronic uveitis or vitritis. For patients with suspected PVRL, non-invasive ocular and CNS evaluations are essential (11, 12). Magnetic resonance imaging (MRI) of the brain and lumbar puncture for cerebrospinal fluid (CSF) analysis constitute first-line investigations to exclude CNS involvement (13). The diagnostic gold standard for this disease is cytology. However, due to its low diagnostic rate, cytokine analysis and gene rearrangement studies have emerged as valuable complementary diagnostic tools (14–17).

Given the difficulty of clinical trials owing to the rarity of PVRL, variable systemic health status of patients, and subspecialist practice patterns, a standard treatment recommendation has not been established for PVRL. Current clinical management strategies include intravitreal chemotherapy, local radiotherapy, systemic chemotherapy, novel targeted agents, and autologous hematopoietic stem cell transplantation.

While external beam radiotherapy (EBRT) demonstrates efficacy in PVRL, it is associated with significant complications, including radiation retinopathy, vitreous hemorrhage, and neovascular glaucoma, alongside a high risk of disease recurrence (18). Intravitreal methotrexate, the most common local therapy, effectively controls intraocular tumors but fails to improve overall survival, prevent CNS relapse, and may induce ocular toxicity (19). Although high-dose MTX(HD-MTX) serves as the cornerstone of systemic therapy for PCNSL, its low vitreous penetration may lead to persistent or recurrent PVRL (20). Furthermore, systemic chemotherapy is frequently complicated by severe toxicities, such as myelosuppression and hepatorenal damage, often necessitating dose reduction or treatment discontinuation.

Lenalidomide, a potent anti-proliferative and immunomodulatory agent, has demonstrated preferential activity against the non-germinal center B-cell (non-GCB) subtype of DLBCL. Rituximab, an anti-CD20 antibody that targets B-cells, has shown significant efficacy in the treatment of PCNSL. Preclinical studies indicate a synergistic effect lenalidomide in combination with intravenous rituximab (R2) in patients with R/R PCNSL or PVRL (8). A prospective study conducted by Zhang et al, 11 patients with PVRL were treated with the R² regimen combined with intravitreal methotrexate, followed by lenalidomide maintenance therapy. At the first evaluation, 10 of the 11 patients achieved a complete response (CR). However, 8 patients subsequently experienced disease relapse (21). This study suggests that while the addition of the R2 regimen to intravitreal MTX induces a high initial response rate in PVRL patients, it is also associated with a high rate of disease recurrence.

Furthermore, CD79B and MYD88 mutations are frequently detected in vitreous samples from PVRL patients (22). These mutations enhance B-cell receptor (BCR) signaling, promoting B-cell activation and survival, thereby representing precise molecular targets for BTK inhibitors (23). As small molecules, BTK inhibitors has been used clinically as a targeted drug for malignant B-cell lymphoma; however, their efficacy in PVRL patients has not been determined. Emerging clinical evidence suggests potential benefit: Guan et al. reported that among 10 PVRL patients treated with ibrutinib, 9 (90%) achieved disease control (DC) at one month, with 7 (70%) attaining a complete response (CR) and 2 (20%) a partial response (9). In a case series by Wang et al, all three patients treated with zanubrutinib maintained a CR with no evidence of CNS relapse across varying treatment durations (24). A comparative analysis by Gao et al. indicated that while the objective response rate (ORR) was similar between BTK inhibitor monotherapy and combination therapy groups (96% vs. 89%), the CR rate was lower in the monotherapy group (79% vs. 92%) (25). These collective findings indicate that combining BTK inhibitors with other therapeutic modalities may represent a promising future direction. An ongoing phase II clinical trial led by Zhang et al. is investigating the combination of Zanubrutinib and rituximab regimen combined with intravitreal methotrexate for PVRL. Preliminary results suggest that this ZR regimen may offer superior efficacy in preventing disease progression compared to the R2 regimen (26).

Orelabrutinib, a next-generation BTK inhibitor, achieves high kinase selectivity and a favorable safety profile through structural optimization, including a reduced spatial angle and fewer hinge-region hydrogen bonds. It demonstrates the highest cerebrospinal fluid concentration (median 21.6–28.7 ng/mL) among BTK inhibitors, correlating positively with efficacy in central nervous system lymphoma (27). In the present case, due to the patient’s poor tolerance to HD MTX, we administered the combination of Orelabrutinib, rituximab, and intravitreal methotrexate, based on existing literature and the patient’s preference. Throughout the six-month treatment period, the patient demonstrated good tolerability without any new adverse events. However, given the limited follow-up duration and the single-case nature of this report, future studies with larger sample sizes and extended observation periods are warranted to further validate the efficacy and safety of this therapeutic approach.

In conclusion, PVRL features diverse clinical manifestations with frequent initial misdiagnosis, and conventional imaging cannot effectively diagnose it or assess treatment response. Dondolin et al. confirmed that ctDNA liquid biopsy combined with PET imaging enables accurate disease evaluation and relapse risk prediction in DLBCL; this approach is also suitable for ocular and other site-specific lymphomas, and its application value in PVRL diagnosis and treatment merits further exploration to underpin personalized therapy (27). This case report provides direct, complementary evidence supporting the feasibility and short-term efficacy of this novel regimen in managing PVRL.

## Data Availability

The original contributions presented in the study are included in the article/supplementary material. Further inquiries can be directed to the corresponding author.
